# Proposed Legislation in Lunacy
*"Report from the Select Committee on Lunatics." Blue Book. Ordered to be Printed, 11th April, 1859.


**Published:** 1859-07-01

**Authors:** 


### Art. IV.—PROPOSED LEGISLATION IN LUNACY.*

The proceedings of the recent Select Committee on Lunatics were brought to a summary conclusion by the dissolution of Parliament; and the Committee have reported that, having regard to this event, as well as in consequence of the inquiry not being complete, "they do not feel themselves in a position to offer any definite opinion on the questions referred to them; and they have therefore agreed to report the evidence taken."

We should gladly, under these circumstances, have deferred any consideration of the subjects submitted to the Committee until the termination of the inquiry; but as the most important portion of the evidence already given is of an official character, we do not feel at liberty to postpone its consideration indefinitely.

We enter upon our task, however, with reluctance, since at the very outset we are compelled to give attention to certain opinions calculated to promote much unprofitable recrimination, and to impede effective legislation for the better care of lunatics. These opinions would not, indeed, have merited notice if it had not so happened that they proceeded principally from the Chairman of the Lunacy Commission, the Right Hon. the Earl of Shaftesbury, and that they influenced several of his suggestions respecting future lunacy legislation. It is to be noted, also, that these opinions are to be regarded as being entertained, in some measure, by the whole of the Commissioners in Lunacy, for, in his answer to query 5, his Lordship says—

> "I would state to the Committee, as well as I can, the present condition of things, and then point out certain amendments which I think might be applied to the existing defects. . . . . Having done that, I should then ask the permission of the Committee to go more widely into the subject, and to point out to them what is the result of my long experience, and the result of the experience of my brother Commissioners, as to the real method of the treatment and cure of lunacy, because we are convinced that it stands at present upon a very vicious principle; and I should wish to indicate to the Committee, and to point out what I consider to be the true and permanent principle which cannot fail of conferring very great benefits upon that enormous class of helpless beings" (p. 1).

It is fortunate, however, for the critic who does not wish to be thrust more than is absolutely necessary beyond the bounds of the subject-proper which he has to discuss, when the author of objectionable and irrelevant opinions himself furnishes the most conclusive answers to those opinions; and this is what Lord

* "Report from the Select Committee on Lunatics." Blue Book. Ordered to be Printed, 11th April, 1859.

Shaftesbury has done in respect to the opinions expressed by him to which we object.

His Lordship was asked, in reference to the medical certificate of insanity (qy. 192)—

"Do you think that there is anything now in the form of the certificate, or what is required to be inserted in it, which creates any unnecessary obstacle in the way of placing persons under that restraint which they clearly ought to be placed under?"

To this he replied:—

"None beyond this, that the certificate declares that the person ought to be taken charge of, and be placed in a lunatic asylum. That at once fixes the taint of insanity upon a family; and we have done all that we could to mitigate the effect. The form used to be, that such and such a person was 'a proper person to be confined;' that was considered painful, and this was substituted, 'to be taken charge of, and placed under medical treatment.' One of the great difficulties arises from this, that you must, in seeking for a certificate, apply, generally speaking, to the medical men in the neighbourhood. Now the knowledge of lunacy among medical men is extremely limited indeed; it has never yet been made the subject of study generally. Of course there are some who have attained to a very great degree of science and knowledge, and there are most eminent names in England at present; but people assume that because a man is a medical man, he must have a knowledge of lunacy; and they therefore apply to him for his opinion; but the fact is, that a medical man has no more knowledge of lunacy than any other human being; unless he has made it his especial study; it is a specialty, and as much requires minute study as anything else. For my own part, I do not hesitate to say, from very long experience, putting aside all its complications with bodily disorder, the mere judgment of the fact whether a man is in a state of unsound mind, and incapable of managing his own affairs, and going about the world, requires no professional knowledge; *my firm belief is, that a sensible layman, conversant with the world and with mankind, can give not only as good an opinion, but a better opinion than all the medical men put together; I am fully convinced of it*" (p. 23).

It is certainly a somewhat novel notion that the necessity of having to obtain a medical certificate creates an "unnecessary obstacle in the way of placing persons under that restraint, which they clearly ought to be placed under." This is scarcely consistent with his Lordship's answer to the 885th query: "In your Lordship's experience, have you not observed that there is a greater tendency amongst medical men to observe the symptoms of insanity than amongst other people?—*No doubt of it.*" (p. 96.) Certainly one of the objects most apparent in the recent attempted legislation for lunatics was to check the supposed evils arising out of this assumed tendency. But we notice this point only cursorily, for it is with the latter portion of his Lordship's answer to the 192nd query that we are, at present, more particularly concerned.

It is not necessary for us to dilate upon the trite axiom that our knowledge of a thing is commonly proportionate to our acquaintance with it; neither is it needful for us to defend the public against the charge of hasty judgment in deeming that the person who has been specifically trained to mark deviations from sound health, whether of body or mind, as is the medical man, is relatively better able than he who has not been so taught to judge of unsoundness whether of body or mind, and to profit by experience. Indeed, his Lordship himself has clearly laid down the prime reason which induces the public to look upon the medical man as the best judge of insanity, for in speaking of the fitness of *examiners* in lunacy being medical men, he remarked, "*because the medical man is the only person* in the neighbourhood of these asylums *who is likely to have given any attention to the matter.*" (qy. 837, p. 94.) That there may be medical men who, notwithstanding the preliminary training mentioned, have profited so little by it as to render their opinion, when their abilities are put to a practical test, of no greater value than the opinion of a man who has had no previous tuition in the method of observing disease, we do not deny; but this is beside the question, yet we may say here, that we fully agree with a hope expressed by his Lordship (qy. 195), that larger opportunities for the study of mental affections will be afforded to medical students in our public asylums, and that these affections will become a more prominent branch of medical study than they now are: but the throwing open of public asylums to medical students is the first step that is requisite. Further, we shall not quibble with the somewhat vague use of the terms "lunacy" and "unsound mind" in the answer, but confine ourselves to a few additional quotations from his Lordship's evidence, bearing upon the opinion that, "the mere judgment of the fact whether a man is in a state of unsound mind, and incapable of managing his own affairs, and going about the world, requires no professional knowledge; a sensible layman, conversant with the world and mankind, can give not only as good, but a better opinion than all the medical men put together; I am fully convinced of it."

In his answer to query 638, his Lordship, referring to the impediments which occasionally occur in removing pauper lunatics to asylums, said—

> "The parish authorities have been desirous to send a patient to the workhouse [? asylum], but sometimes the medical man will not certify; and in several instances they have gone before the magistrates, *who know little or nothing about it*, and they have said 'This person appears to be much too tranquil to be sent to a lunatic asylum; I will not sign an order.' *And there is the evil of being obliged to take them before persons who know nothing about the matter*" (p. 72).

Again, Mr. Tite having asked (qy. 846), "Would your Lordship approve of a pauper lunatic being taken before a magistrate that he might see the case?" the reply was—

"The honourable member means, I suppose, private lunatics. I think nothing could be worse than that; there would be a degree of publicity about it that would be most painful, to go before a magistrate, and to have the matter determined by him, whether the patient should or not be put under medical treatment. *In* 99 *cases out of* 100 *the magistrate knows little or nothing about the matter.* A case occurred the other day, of a poor man, who was taken before a magistrate, and he refused to certify, because the man was not in an infuriated state. 'A quiet person like him,' he said, 'ought not to be put in an asylum; take him back.' He was in a low, desponding state; and if he had been sent to a curative asylum he might have been cured and restored to society" (p. 95).

If we now turn to query 838, we shall find that his Lordship entertains as little respect for the opinion of a clergyman on a case of lunacy as for that of a magistrate. His Lordship was questioned concerning the propriety of having "medical examiners" to countersign and confirm the medical certificates which authorize the reception of a patient into an asylum, and thus afford an additional protection against unjust confinement. As the answers to queries 836 and 837 have an immediate bearing upon our present topic, and upon the answer to the 838th query; and as one reason advanced for selecting a medical man as an examiner, if such an officer should be appointed, is curious, we shall quote the answers to the 836th and 837th queries as well as the answer to the 838th.

"836. If this duty were imposed upon the Commissioners, would it not be performed by the medical Commissioners?—No; it might be done by them, but there again comes in the objection which I so strongly entertain, and I protest against bringing the medical profession so forward that they only are to be the judges of insanity.

"837. Does not that apply to the clauses of this Bill, that require that the examiners shall be medical examiners?—Yes; *because the medical man is the only person in the neighbourhood of these asylums who is likely to have given any attention to the matter.* You could not impose upon the magistrates or the resident gentry such a duty, but it must be put upon a person who is generally resident, *who will take a fee for his duty, and a medical man is almost the only person to do it.*

"838. *Mr. Whitbread.*] Could not you attain this object by insisting by law upon some religious care of the patients by some chaplain who could equally form a judgment?—*I should have more distrust of the religious gentleman than I should have of the medical man; and I say*

*that with the deepest respect for the ministers of religion.* The difficulty of it would be incalculable if you were to throw the duty on the parochial clergy in the neighbourhood, who are already over-burdened" (p. 94).

Now, in his reply to the 194th query, his Lordship says, "*It is clear that the value of the certificate* [of insanity] *must depend upon the experience of the people who sign it.*" (p. 23.) But in a subsequent portion of his Lordship's examination we read as follows :—

"867. Clause 15 [of the Bill introduced by Mr. Walpole 'to amend the Law concerning the Care and Treatment of Lunatics'] is, that 'certain persons are not to sign certificates for the reception of patients.' That is where they are the proprietors of any licensed house ?—Yes.

"868. I believe your Lordship considers that a very important clause ?—Yes ; although I believe very great objection will be taken to it by the medical men.

"869. Your Lordship approves of that clause ?—Yes.

"870. That clause will prevent their playing into each others' hands ?—Yes;" &c. (p. 97).

Thus the only section of medical men who have, with few exceptions, an opportunity of becoming thoroughly experienced in insanity, and of bringing that experience to bear upon the public good, are at once swept aside, and the signature of those important documents, the medical certificates of insanity, left entirely to that portion of the profession least practically acquainted with the subject. For it must be remembered that the medical officers of public asylums are debarred from private practice entirely.

If, then, we sum up his Lordship's opinions on the first obnoxious point that has demanded our attention, we find (1) an assertion of the comparative worthlessness of the opinion of most medical men on cases of lunacy; (2), an expression of "firm belief" that the opinion of a sensible layman on such cases is of greater value than that of a medical man ; (3), an assertion that the opinion of a magistrate is valueless from his knowing nothing whatever about the matter ; (4), another assertion that the opinion of a clergyman is even of less value than that of a medical man ; (5), a statement that an opinion depends for its value on the experience of the person giving it ; and to crown all, (6), an approval of a suggestion by which the bulk of the most experienced practitioners in lunacy in the kingdom would be debarred from uttering an opinion at all in the form in which a medical opinion on lunacy is of the greatest importance, to wit, when expressed in a certificate of insanity.

We shall not attempt to reconcile these apparent contradictions, but add, as a pendant, an answer which would seem to imply, that his Lordship is disposed to look with confidence upon the opinion of a *lawyer* alone in cases of lunacy.

Mr. Monckton Milnes asked—"Is there any advantage in these medical examiners being medical men; why not lawyers?" To which his Lordship responded, "*I would not object to that* if we could find a person in the neighbourhood who, being resident, *would take a fee for it.*" (p. 96). Mr. Milnes must have been in a somewhat waggish mood, and disposed to pleasantry, when he asked if there would be any advantage in the *medical* examiners being *medical* men, and then suggested *lawyers!!* .

The second obnoxious point that we have to deal with is as follows:—

"His Lordship feels strongly that the whole system of private asylums is utterly abominable and indefensible." (Qy. 101, p. 14.) On this question he explains himself more fully in his answer to query 494—

"It is the result of very long experience in these matters, that a large proportion of the difficulties in legislation, and almost all the complications that we have to contend with, or to obviate, arise from the principle on which these licensed houses are founded. The licensed houses are founded upon the principle of profit to the proprietor, and the consequence is, that any speculator who undertakes them having a view to profit is always eager to obtain patients, and unwilling to discharge them; and he has moreover the largest motive to stint them in every possible way during the time they are under his care. Now, this must be borne in mind, I do not intend to cast any reflection upon the medical profession. I know that when I have urged arguments of this kind, I have been told that I have entertained most undue suspicions of that great profession. I have no suspicion of them as medical men; but my suspicions are of the medical men only when they are proprietors of lunatic asylums, into which lunatics are taken for profit" (p. 54).

Again, his Lordship states further, in his answer to query 504—

"I know that there are some asylums extremely well conducted; I know that nothing can be more attentive, more minute, or more conscientious than the care that some of these proprietors take, but we have no security; they are here to-day, and they may be gone to-morrow. True, there are some very good men; and perhaps we might be content with what we have, and not endeavour to effect any alteration; but the licence by the death of one proprietor may pass into the hands of another, and he might act upon totally different principles: and you have ever to contend with that vicious principle of profit. Now, if you read our Acts of Parliament, it will be seen that half of the provisions are made to enable the Commissioners to fight against the selfishness of persons who open these asylums. After perpetual anxiety and trouble, we may manage to get an asylum into a decent condition; and while we are in the presence of this vicious principle, we keep it down. We direct certain things to be done in a house, and very often there is an appearance of their being done; but when we turn our backs, that principle which we have curbed by our presence recommences its active operations; and we cannot have any security whatever that justice will be done to the patients, because we cannot dog the thing day by day, or hour by hour, and know that every condition is fulfilled. Where a proprietor is unprincipled, see what advantages he has, and what power he has over his patient. It is in vain to trust to the case-book; for, as I read in a letter from a medical man the other day, he says that the case-books can be and are very frequently 'cooked.' When a certain diet, for instance, is prescribed, what security is there that that diet is given? We know that a certain course of medicine is necessary; but what security is there that that course of medicine is undergone by the patient? And it is therefore in their power to retard the cure of the patients indefinitely, and the temptation is inordinately great; and it is more than human nature can ordinarily stand. There are some cases in which the patients are paying from 400*l.* to 500*l.* and 600*l.* a year; and the loss of one or two of those patients would be a dead loss—a loss of the most serious kind—and one that would fall very heavily upon the condition of an establishment; for the proprietor is by no means secure that another patient, able to pay an equal amount, will come to take the place of the one he has lost. I remember one instance, not very long ago, where a patient was paying no less than 1200*l.* a year; and I am certain that the expense of that patient in the house was not 300*l.* a year; so that was 900*l.* a year clear profit to the medical man. You sometimes see it paraded that such and such a patient has the benefit of a carriage. I do not much believe it. It sometimes happens that they get a drive when the doctor has taken his drive; but as to the permanent use of a carriage, I am sure that very rarely happens. But in that case, where 1200*l.* a year is paid, I say that there is the strongest temptation to retain that patient. It is not necessary to be under such influences, that the medical man should be a person of actually dishonest intentions; but when there is temptation such as that, can the Committee not imagine all the self-delusions that a man would practise, and the disaffection with which he would look upon any returning symptom of health? how he would consider that the matter required further consideration, and so retard the period of the discharge, if it ever took place? I am certain that the temptation is so great, that few people could resist it. I do not believe that any person could, in fact, resist it. *I am certain that I could not resist it; and therefore I feel very strongly that some control or check must be put upon the present system*" (p. 56).

Of course, the frank statement of his Lordship, that if he were similarly circumstanced to the proprietor of a private asylum, he could not resist the temptation of making as great a profit as possible out of his patients, without regard to their welfare, at once puts a stop to serious argument upon his assertions; but we must demur to the conclusion that, *because* he unfortunately could not resist temptation, and might not be (as Sir Erskine Perry phrased it in a subsequent question respecting proprietors of asylums) "actuated by the ordinary motives which actuate human conduct, viz., to succeed with his patient, and thereby give evidence of his skill and ability," therefore it was necessary to have additional legislative control over the present system of private asylums. We may, however, add, that we should have a greater confidence in his Lordship's probity under temptation than he himself seems to entertain; for even admitting that (as his Lordship would soften the matter) a man may slide almost unintentionally into wrong, the act is, after all, one of rank and indefensible dishonesty, and one that could not suggest itself for a moment to a pure-minded, honourable, and Christian man. The Earl of Shaftesbury, we believe, greatly under-estimated his own moral powers of resistance when he made this statement.

As to the positive statements made in the answer we have just quoted, but few remarks are requisite. How does it happen that so serious a matter as the falsification of case-books is mentioned by the Chairman of the Commission in Lunacy upon the vague authority of an unnamed medical man? The Commissioners have the power, and it is their peculiar duty, to institute inquiries into charges of this kind. Why was no inquiry made into the charge paraded by his Lordship, or if made, why were not the results laid before the Committee, and not a vague and, in the mode in which it was put, unwarrantable charge? Again, of the case mentioned by his Lordship as paying 1200*l.* a year, he says,—"I am certain that the expense of that patient in the house was not 300*l.* a year; so that was 900*l.* a year clear profit to the medical man." But in his answer to query 914, his Lordship states, doubtless speaking of the same case, "I have in my mind an instance where a lady paid 1200*l.* a year to the proprietor of an asylum, *and I am quite sure, when we visited her, the sum expended upon her did not amount to* 400*l.*, leaving a clear 700*l.* or 800*l.* a year profit." (p. 100.) These statements might be very well left to tell their own tale, but we will add that, as the Commissioners must know, when payments of unusual magnitude are made for the care of patients, they usually represent the magnitude of the responsibility involved in the case, and of this responsibility and its money-value the friends of the patient may be conceived to be the best judges. Moreover, the Commissioners have no difficulty in obtaining the fullest information respecting the treatment and care of private patients; and Lord Shaftesbury might, if he had wished it, have obtained the minutest knowledge of the *cost* (apart from all estimates for professional emolument) of the case he referred to, and thus have ascertained how ridiculously erroneous his own estimates are. It is no explanation of the course his Lordship took, that he could not place any confidence in returns made to him by interested parties; for it is evident that his own statements are simply guesses.

Now, we protest against opinions and statements such as those we have quoted from the evidence of Lord Shaftesbury being obtruded into the midst of a grave and important inquiry. That they are unsupported by any evidence worthy of consideration, and that they are calculated to impede effective legislation for the better care and treatment of lunatics, by the substitution of *surmises*, and of partial and unguarded statements of facts for carefully ascertained data, thus leading public opinion astray, is evident. And if mere suspicions of any class of men are to take the place of serious argument, where shall we draw a limit to the exercise of this double-edged and indefensible method of reasoning, if reasoning it may be termed?

We have but to turn to another portion of the evidence given before the Committee, to ascertain to what it would tend.

Mr. Bolden, the honorary solicitor to the Alleged Lunatics Society, being asked, respecting asserted wilfully inaccurate copies of medical certificates of Lunacy—

"Have you any copy of that kind, or can you produce any certificate which was so inaccurately copied, that if the original had been taken by itself it would not have justified the reception of a patient, but if taken with the copy it was so altered as to justify that reception?" He replied, "I cannot. The only certificates that you can ever see are the copies that are obtained from the Commissioners in Lunacy, and *those copies the Commissioners take care are quite sufficient to justify the detention.*" (Qy. 2674, p. 222.)

Again, Admiral Saumarez, the chairman of the Alleged Lunatics Society, being asked, in reference to the power possessed by the Commissioners of granting permission to visit patients in private asylums,—"Do you mean that any person who wished to visit a patient within the metropolitan district would not have full opportunity of going to any of the Commissioners and ascertaining from them whether such a visit might be reasonably allowed or not?" replied, "Yes; *but they are not altogether an independent party, and it would be almost a charge against their surveillance to suppose that any individual is confined improperly without their knowledge in an asylum*, and therefore I would give the public some further protection than the Commissioners in Lunacy. *I do not think the Commissioners in Lunacy are fit and proper persons to have that power, because, as I have already stated, they are interested parties.*" (Qy. 2785, p. 231.)

Let us now give attention to the more important portions of Lord Shaftesbury's evidence, avoiding as far as practicable the sequences of the opinions we have quoted, and premising that a considerable part of the evidence consists of full details of the duties of the Commissioners in Lunacy, the mode in which they are carried out, and the enactments under which they are performed.

One of the first points of general interest upon which his Lordship was questioned was, the source of the great increase in the number of *known* lunatics which had taken place between 1845 and 1858. Did this increase arise from an actual increase in the number of lunatics in the kingdom, or was it apparent only, and dependent upon the greater provision made for, and the greater care given to lunatics, during the period named, in consequence of which many previously unknown and unrecognised cases were brought to light? His Lordship is inclined to answer in the affirmative; but, in addition, he expresses the opinions that, (1) as regards the pauper classes, while he believes that there is an actual increase of insanity among them due to increase of population, yet that that increase is not "by any means in proportion to the increase of population;" and (2) that as regards "persons a degree above pauperism," and "the classes beginning with the trading classes and persons keeping small huckster's shops, and rising to the highest vocations in life," he cannot "but hazard the opinion," to use his own expression, "although I dare say many will differ from me, that if there is not an actual increase of insanity, there is developed a very considerable tendency towards it." (Qs. 49, 51, 59.)

Mr. Gaskell, one of the Medical Commissioners in Lunacy, is inclined to attribute the increase in known lunacy to the prolongation of the life of lunatics in our asylums, in which "the annual mortality was perhaps 12, 14, or 15 per cent. formerly," but "it is now reduced to 10 per cent." (Qs. 14, 36.)

There can be little doubt that the causes assigned by Lord Shaftesbury and Mr. Gaskell have contributed greatly to the increase of known lunacy in the kingdom, but we are still left in the dark as to the extent to which these causes have influenced the increase, and until this be determined we cannot, with our present data, decide upon the increase or not of insanity, either among the population at large, or among any section of the population. Hence Lord Shaftesbury's speculations upon the rate of increase of insanity among paupers, and the state of insanity among the classes of the population above pauperism, are entirely hypothetical. His Lordship is, however, thoroughly aware of the doubtfulness of his opinion upon the increase of lunacy in the country, for he says—"I am almost afraid of giving an opinion, as it may be the commencement of the most awful controversy, for there is a great difference of opinion on that point, because all the data preceding the year 1845 are so very indistinct, and even subsequently to that, they are so few, and so mixed up with all those old chronic cases, that it is difficult to say what has been the increase." (Qy. 51.) Under these circumstances we deeply regret that his Lordship did not content himself with pointing out to the Committee the defective state of our present statistics of insanity, the impossibility of determining the important question of the increase of insanity or not until the defects had been removed, and what was wanting for the removal of the defects.

The approach of the Census of 1861 at once directs attention to the only method by which an accurate knowledge of the amount of insanity, and an accurate basis for the calculation of its progress in the kingdom, can be obtained, and we had hoped to have heard this urged to the Committee, and to have witnessed his Lordship's influence, and that of the Commission in Lunacy, brought to bear in bringing about the sole possible mode of obtaining proper data for solving a much debated and exceedingly important question—a question, indeed, of growing importance, both scientifically and practically.

In a subsequent part of his examination, Lord Shaftesbury expressed the opinion, when speaking of the untrustworthy character of the records we possess relative to the mortality and recoveries of the insane as a means of comparison concerning the character and results of different asylums, that—

"It would be very desirable if we could have proper statistics on insanity drawn up and put upon a good footing. It would require great trouble and expense; but I think it would be worth the trouble and expense, if it could be put into the hands of some competent persons; and I have no doubt that some most remarkable results would be brought out. In our department we have got a great deal too much to do; the Commissioners are constantly at work, and the clerks too." (Qy. 263.)

This is so far well. But we do not want remarkable results; we want the ordinary A B C results of insanity statistics which we do not yet possess. We do not want, morever, a huge, expensive inquiry into existing statistics; that would be of little or no value. We want a scheme of insanity statistics which might form a portion of the ordinary returns, and govern many of the returns, to the Commissioners; which would take its proper place in the legitimate working of the Commission; and which for the future would furnish us with trustworthy and regular information. Such a scheme would have to emanate from a qualified worker in statistics; it is quite practicable, and might be put in operation without extraordinary difficulty; and it would doubtless require little other expense, for its regular carrying out, than would be involved in an additional clerk to the Commission. Let a scheme of this kind be developed, and let us obtain a basis of knowledge respecting the insanity of the whole kingdom in the next Census, and we may look forward to the probability of being able, at a comparatively early period, to avoid the slough of vagueness into which every one is plunged who ventures to meddle with insanity statistics.

The want of reliable data reduces also, very considerably, the value of his Lordship's opinions on the causes of insanity. He attributes "one-half and perhaps more" (Qy. 51) of the cases of insanity among the poorer classes to intemperance, and quotes several medical authorities in support of the opinion that intemperance is a powerful cause of insanity. He also seeks to explain the assumed decreasing rate of insanity among the poor, by further assuming a decrease of intemperate habits; but it will be early enough to discuss this point when the prior assumption is proved to be worthy of credence. It is also unnecessary to seek with his Lordship, in the excitable state of society among the classes above pauperism, a cause of an increased tendency to, or actual increase of, insanity, until the one or the other is shown to exist. As to other causes of insanity, general or special, we must quote from the evidence.

61. *Sir George Grey.*]—After deducting 50 per cent. of the cases which are attributable to drunkenness, is there any other one prominent cause by which to account for the insanity of any large proportion of the other 50 per cent. ?—There is sometimes a strong hereditary predisposition ; a good many come from accidents and blows upon the head.

62. You cannot indicate any one cause to account for any large proportion of the other 50 per cent. ?—No.

63. I presume they would be attributable to a great variety of causes ?—Yes, but I do not remember any one cause of equal importance to that of intoxication.

64. *Chairman.*]—What is the predominant cause among the richer class of lunatics ?—It appears to be a disordered imagination, hereditary predisposition, the pursuit of money, disappointed ambition, or great losses in trade, and sometimes you will find it from over-work.

65. *Mr. Tite.*] And from religious excitement ?—No. Am I to understand the Honourable Member to ask whether religion is the cause of madness ?

66. *Mr. Tite.*] Yes ; enthusiasm ?—

67. *Chairman.*] Have you any tabular statement that will show the different causes of insanity, and the proportions of the cases arising from those causes ?—We have papers ; we have a full account of all the patients, and a full account of the causes, so far as we can ascertain, of their madness.

68. Are these accounts analysed and arranged ?—No ; but we could furnish them to the Committee.

69. *Mr. Tite.*] My question did not mean religious excitement in the ordinary sense of the word, but different classes appear to me more divided upon religion than they were; has that, in your opinion, led to insanity?—That is one of the most important questions that can be put, and I am very glad that the Honourable Member has put that question, because I think there are two or three observations to be made upon it, which may tend to remove a good deal of misunderstanding upon the subject. I, of course, should have very great diffidence in speaking upon this question, if it were purely a medical question; but it is a moral question, and therefore any non-professional person, any layman who gives his mind and heart to this subject, has as much right to speak upon it as all the physicians put together; and I do not hesitate to say, as the result of my experience, that I have never seen a case, and I have never heard of a case, in which a person has gone mad caused by the influence of religion; and when I say by the influence of religion, I mean the true Gospel spirit of true Christianity. I do not mean that a person may not have been turned aside by some strange notion, that some ignorant timid person having taken up some obscure and mysterious point of religion, and looking at it constantly and exclusively, may not have become disordered in his reason; but religion, taken as the pure Gospel, I will never believe has had the slightest effect in producing any aberration of reason whatever.

Now we are prepared to go with his Lordship hand and heart in any raid he is desirous to make upon intemperance, but we are not disposed to lay aside prudence in so doing. If the statistics of insanity would aid us in scotching (we do not hope to kill) the evil, we should gladly avail ourselves of them, but still decline to rush so hastily with them to the front as to leave our flank and rear entirely exposed. We are inclined to attribute a very important place to intemperance as a determining cause of insanity, but if we were to elevate it above hereditary influence, or to give it the prominent position which Lord Shaftesbury does, and make it the remote cause of the predisposition to insanity among certain classes of the population (Qy. 52) as well as the primary exciting cause, we should fear to weaken our argument by stating what we could not satisfactorily support, and thus lay ourselves open to disregard when we sought for respect. But why, upon a subject of such great interest, should we have any unnecessary vagueness, if, as his Lordship states, the Commissioners in Lunacy "have a full account of all the patients, and a full account of the causes, so far as we can ascertain, of their madness?" Why should his Lordship have contented himself with a doubtful, and, indeed, in great measure speculative answer, and refer to data collected many years ago, when the question touched upon the present time, and the materials for a tolerably positive reply were accessible to him? It is to be regretted that the Committee did not ask for the return proffered by his Lordship, and we trust when again called together they may do this, for an account of the *assigned* causes of insanity throughout England and Wales for a definite period would be of great interest.

Not having the clue to what Lord Shaftesbury means by "the true Gospel spirit of true Christianity," we cannot enter with any benefit into the discussion which he starts respecting the influence of religion as a cause of insanity. The signification of the term religious enthusiasm, or religious exaltation, as used by medical men to express an occasional cause of insanity, is tolerably well understood, and is not usually supposed to convey any disrespect of religion. Perhaps the best commentary upon Lord Shaftesbury's remarks on the causes of insanity is a return made by the French Asylums in 1853, and recently published among other returns relative to insanity in France, by the Imperial Government. We quote M. Brierre de Boismont's summary of the most important points of the return referred to, partly for brevity's sake, and partly for the additional remarks on the value of asylum returns on the causes, and on intemperance as a cause, of insanity.

"Of 2883 lunatics in 1853, hereditary predisposition is stated to have existed in 1410 men and 1470 women. Of 1000 lunatics, the cause of insanity was said to be physical in 572, and moral in 428. We have already remarked upon the necessity of living in intimacy with the insane, in order to obtain a correct knowledge on this subject; and that the inexact information obtained in asylums, both public and private, reduces very much the value of the figures referring to heritage and other causes. There are also other objections in regard to the physical causes, because it is evident that drunkenness, bereavement, and misery have a double meaning. The man who, for example, drinks to stupify his grief and becomes insane, at first acts under the influence of a moral cause. Accidental suppression of menses (150) and puerperal insanity (150), in a great number of cases, arise from moral impressions.

"Among moral causes, the most frequent is grief arising from the loss of money; 899 cases of insanity are attributed to this cause, which is, by comparison with the total figure of moral causes, a proportion of more than 12 per cent. Afterwards come religious exaltation (894), love (792), violent emotions (698), pride (600), the loss of a loved one (510), disappointed ambition (495), jealousy (442), political events (308), excess in intellectual work (156), *simple* imprisonment (154), nostalgia (48), isolation and solitude (41), change of life (32), association with and assiduous attention upon the insane (16), *cellular* imprisonment (4)."*

In the official returns, out of 9764 cases assigned to physical causes, 2594 are attributed to epilepsy and convulsions, and 1502 to drunkenness.†

* "Journal of Psychological Medicine." No. XIV., N. S. p. 304.

† "Statistique des Etablissements d'Alienes de 1842 à 1853 inclusivement," 1857, p. xxxix.

this quote

We have already quoted a sufficiency of Lord Shaftesbury's evidence on private asylums to show with what feelings of animosity he regards those institutions. It is now needful that we should know what suggestions he makes for the diminution or removal of the evils which he regards to be all but inseparable from them. We may state first, however, that his Lordship is of opinion that "the state of licensed houses, and the condition of the poorer patients" in them, have been "*inconceivably*" improved of late years (Qy. 157); and we may also remark that he also states that the chief obstacles to their thorough reform (apart from the "principle of profit") are constantly diminishing (Qy. 103).

Notwithstanding that Lord Shaftesbury is disposed to regard a medical man's *opinion*, as a general rule, of no greater value than that of a sensible layman, yet it is requisite to name that, in so far as the *care* of the insane is concerned, he considers that the medical man should be all-powerful. Thus his Lordship being asked, "Is it not the fact, that in the case where the medical man is under the proprietor, he is, in fact, liable to be very much influenced by the proprietor of the asylum?"—he replied, "There is no doubt about it; and it is not the true position that a medical man ought to occupy. The medical profession stands too high to be placed in that position; and they are under influences which they cannot resist. I have reason to speak in the highest terms of estimation of some of the medical men in charge of asylums; and I can only deeply deplore that they are not placed in their true position" (Qy. 94). Again, being asked whether (Qy. 95) "The medical man ought to be a kind of inspector and check upon the proprietor?"—he replied, "And more than that; he ought to be lord paramount in the asylum."

Badly as Lord Shaftesbury thinks of private asylums, and vicious as he consider the principles upon which they are worked, he would not, however, do away with them altogether; but he would diminish their operations as far as practicable, by the establishment of self-supporting asylums for the middle and higher classes of society. But it is necessary to quote his Lordship's evidence upon this point—

"507. * * * * * I now speak with reference to that large class of society which begins just above pauperism, and goes on to the highest in the land. All the difficulties in legislation arise out of that particular class; we have none with respect to the management of the paupers in the county asylums; they go on very well. There is nothing in them but the ordinary decay, and the difficulty that arises in all institutions out of occasional ignorance and mismanagement. If you had establishments of that kind, asylums or public hospitals, I should like to say chartered asylums, you would find that they would be precisely the reverse of those I have mentioned. First of all, there would be a total absence of that motive which constitutes the vicious principle of the present licensed houses, there would be no desire or view to profit of any sort.

"508. *Chairman.*] Do you suggest that there should be no private asylums of any sort or kind, and would you absolutely prohibit them by law?—No, certainly not. I would leave them, and leave it to the public to choose which kind of asylum they wished to go to. I have no doubt that a certain number of those licensed houses would continue, and I dare say that persons, from peculiar notions of their own, would resort to such asylums. I would allow them to continue, and I would also have, as you have public asylums for paupers, houses on a public footing for persons in a better condition of life.

"509. Would you have them in every part of the kingdom, as you have public asylums for paupers?—Yes. These asylums would be quite free from all those vicious motives that have been referred to in the licensed houses. There would be no eager desire to take the people in, and no desire whatever to stint them in any one way, and so far from there being a desire to retain them, they would run rather into the other extreme, for there would be rather a desire to turn them out; because I think there would be such a pressure upon them that their only anxiety would be to make room for others by turning some out.

"510. *Sir George Grey.*] Before your Lordship fully states the results which you anticipate, will you define how they will be maintained, whether at the public expense, or from local funds, and under what control they will be placed?—I will. I was going to add that these asylums would be free from all the objections which exist to licensed private houses. The example which I principally should follow would be the example of Scotland. In Scotland, the chartered asylums have existed for a certain number of years, and they have been productive of the very greatest benefit. We have a certain number of institutions similar to them in England, and they are called hospitals. Hospitals in England are founded upon private funds. The chartered asylums in Scotland are also founded upon private funds. I do not think that any one of them, except that at Morningside, has ever received a grant, and I think that it received 2000*l*. out of some of the forfeited estates in the year 1745, and to what extent they have been considered satisfactory to the people of Scotland I will show: of 833 private patients, placed in asylums in Scotland, 652 are in chartered asylums, and only 230 in licensed houses. The contrast in this respect between Scotland and England is very striking. By the last Report of the Commissioners in Lunacy, it appears that there were 4442 private patients in asylums in England and Wales; of those, 2746 are in private asylums, and 1696 are in public hospitals; these latter comprising 669 patients in Bethlehem, St. Luke's, and Guy's Hospital, and the Institution for Idiots. Then it goes on to show how popular they are, and how beneficial they have been, in every sense of the word, and how they have provided the very best care and treatment of the patients, and that it has been done at a very low figure compared with the cost of other establishments.

"511. You have not stated where the funds come from?—Those are founded upon private contributions.

"512. *Sir George Grey.*] Sustained by private endowments?—Yes.

"517. *Chairman.*] What you wish for is an encouragement for the endowment of hospitals for lunatics?—Yes; to be founded in two ways, either, as in Scotland, and in some parts of England, by private contributions, and we have 11 hospitals in England also so founded, or, as in England, in respect to borough and county asylums, upon the public rates. I think it would be sufficient only to make known the want, and I have no doubt the money would flow in: but I would give in the Bill a permissive clause to counties for the purpose of founding these asylums entirely for the reception of the middle class patients.

"518. *Mr. Drummond.*] Do you mean to be annexed to others?—Not necessarily so.

"519. *Sir George Grey.*] A permissive power to found them out of the county rate?—Yes; it would not require that the county should do more than give the guarantee of its rates; it would not be necessary that the county should expend a farthing, in fact it would incur no hazard of its own whatever. But then it should have a power to erect an asylum of that description; I would leave the governing power, the initiating power, just the same as with regard to the county asylums, with the magistrates in quarter sessions, or it might be vested in the visiting justices of the present county asylums, who, having considered all matters, might, with the consent of the magistrates in quarter sessions, if they thought it desirable to institute such an asylum, merely take the guarantee of the rates, to raise the necessary sum of money upon the guarantee of the rates, and raise the money at 6½ per cent., the whole interest and principal being thus paid off in 30 years. The thing would be self-supporting, and the moment the asylum was opened it would be filled with patients, some of a higher class and some of an inferior class, who would pay the whole expense; their payments would cover not only the 6½ per cent., but the whole expense of carrying on the institution, the care and maintenance of the inmates, and all the salaries and everything else.

"520. Might not that interfere with their being founded by voluntary contributions like hospitals for any other disease?—The voluntary principle has its limits, and I think that the voluntary principle on this head has reached its utmost limits in England. It has founded 11 hospitals that have worked well, but the voluntary principle has not gone any further, and I do not think it is likely that any more will be founded."

His Lordship contemplates that the cost of keeping a patient in one of these institutions might be reduced to 15*s.* a-week, the cost in a private asylum being from 35*s.* to 40*s.*; that with this diminished cost all the benefits of a well-managed public asylum might be obtained, and the disadvantages and evils of a private one avoided; and that these advantages would be placed within reach of persons who cannot afford to pay the costs of a private asylum, and yet wish to avoid the humiliation of consigning a lunatic relative or friend to a pauper establishment.

Dr. Conolly's evidence upon chartered asylums will form a fitting note to Lord Shaftesbury's suggestion.

"1956. Have you considered the system that is pursued in Scotland in the chartered asylums, basing these asylums for the middle classes on a public foundation as they do ?—Yes, I believe that some of them are very excellent institutions, but I do not very much approve, indeed, I more than doubt the propriety of having patients belonging to the higher classes and pauper patients in the same institution.

"1957. You think it would be desirable if public institutions for the middle classes could be established in this country ?—Most desirable.

"1958. To supersede the private asylums altogether ?—Not exactly that; I mean for persons who cannot afford to pay, say 100*l.* or 50*l.* a year. There is scarcely a week in a year in which people do not apply to me either about a daughter, perhaps a governess, or a young man with a small salary, and they cannot pay the necessary expense of the cheapest asylum, and the friends are reduced to ruin by it; it often happens in the families of the clergy, and such cases have occurred to me over and over again.

"1959. *Chairman.*] In that class of cases no asylum could pay its own way without voluntary contributions ?—I believe none; there are but few asylums where we can now send them like Northampton and Coton Hill; but many more such institutions are very much wanted.

"1960. *Sir George Grey.*] The Committee have been informed that there are eleven such now existing in England. Have you had any personal experience in the management of those institutions ?—Yes, I have seen something of the one at Northampton, and a little of that at Coton Hill, near Stafford, and I have the highest opinion of both.'

It is simply necessary to add to this quotation that, while Dr. Conolly points to a want which our private asylums cannot, and our public asylums do not, provide for, but which might be obviated by the establishment of asylums capable of meeting the requirements of persons of contracted means; Lord Shaftesbury seeks to provide, in the first instance at the public cost, asylums which would accommodate alike those of contracted and those of abundant means. The one scheme would probably be a feasible plan to attain a well-defined and clearly requisite object; the other, while including this object, would aim also at one the necessity for which has not been shown.

The next topic which demands our attention refers more or less to the medical certificate of insanity which is requisite before a lunatic can be confined in a private asylum.

The Chairman of the Committee put the following question (qy. 185) :—

"The medical man, if he be the proprietor of the house, has a direct interest, if the patient be rich, not to part with him. If there is no doubt about the facts, which is the most important part of the whole proceeding, is it not necessary that there should be some authority who might be put in motion by the relations independent of the medical superintendent of the house, who might be interested in keeping a patient in, in order to see the Commissioners were rightly informed with reference to the case of a patient who was confined?"

To this Lord Shaftesbury replied:—

"If you could devise such a personage as that, it would no doubt be beneficial, but you must be careful, while you are endeavouring to protect the patient, that you do not throw too great impediments in the way of his being put under proper care; for, not only are the public to be protected, but even in the interests of the patient you must not multiply the difficulty in determining the point when a person should be deprived of his natural liberty, and be subject to restraint and medical treatment. If you wait until the symptoms are so clear and so developed that there can be no doubt about it, then you will have waited till such a time that the man is probably become an incurable patient; but if the case be taken in time, when the symptoms are only discernible to an experienced eye, and when they would not be discernible to an inexperienced one, the probability is that the man will be cured, and will return to society in the course of a very short time. It is a curious thing, but it is the result of very long experience, and I am sure the Committee will find that all my brother Commissioners will concur in it, *that however imperfect the certificate is, in a great variety of respects, remember in the first place it is the only document that you have to go on, and therefore you must, to a very great extent, rest upon it; but nevertheless there are very few instances; I do not think, in my experience of nearly thirty years, a single case, or not more than one or two cases, in which any person has been shut up without some plausible grounds for his or her temporary confinement; but in every instance, with these exceptions, there have certain plausible grounds in facts and in logic, sufficient to justify the temporary confinement of the persons and their being submitted to medical treatment.* I believe that very few have been really shut up without cause, but I have no doubt that very many indeed have been detained beyond the time when they might have been set at liberty; I hope, however, that we are reducing the number every day, yet I have no doubt that many are detained a long time beyond the period when they should be set at liberty.

"186. *Mr. Tite.*] But who were not received improperly originally?—No.

"187. *Mr. Coningham.*] Do you mean detained by interested parties?—Yes, detained by proprietors, or detained through the non-intervention of their friends; but such is the melancholy condition of patients, that from the moment a patient is struck by this affliction of Providence, from that hour he becomes, civilly and morally, dead in respect to his relatives."

important

We presume that no one will question his lordship's position respecting the inadvisability of throwing unnecessary impediments in the way of the early treatment of lunatics, yet it is to the persistent misapprehension of the efforts made by medical men to bring about this early treatment, that several of the most important difficulties of lunacy legislation are due; and this must be so if, as in Lord Shaftesbury's evidence, mere suspicions of the most offensive character are to be permitted to take the place of facts, and to overlay all that the medical proprietors of asylums, and medical practitioners in general, have done for the amelioration of the care and treatment of the insane. "If you wait," says his Lordship, "until the symptoms are so clear and so developed that there can be no doubt about it, then you will have waited till such a time that the man is probably become an incurable patient." But *who* is to decide upon the incipient symptoms of lunacy necessitating treatment, if, as his Lordship asserts (with what consistency we have already seen), *the opinion of a sensible layman upon the symptoms of lunacy is of greater value than that of a medical man* (Qy. 192); or if his Lordship's views concerning insanity are to be taken as our guide.

"193. *Mr. Coningham.*] Insanity is always accompanied, is it not, by a morbid condition of the brain?—It may be so or not; still it is not always apparent. Insanity is often accompanied with bodily derangements and symptoms which the medical man alone can deal with; but it is not in all cases the object so much to determine whether a man is out of his mind or not, as to tell whether a man, although being a little queer, as it is called, is capable of managing his own affairs. A man is not to be shut up because he is eccentric, or somewhat strange. If a man is altogether harmless, capable of taking care of himself, and of managing his own affairs, and not in an early stage of the disorder, there is no reason why that man should be shut up.

"194. But insanity is invariably accompanied, is it not, by a morbid condition of the brain? — I am afraid of going into that question. Many men say that there is such a thing as moral insanity, which is not connected with any functional disorder. However that may be, it is clear that the value of the certificate must depend upon the experience of the people so sign it."

If the dicta contained in the answer to Query 193 are to guide us, of course the early treatment of cases of insanity would be rendered an impossibility, and an asylum would become solely a house of detention for chronic and incurable lunatics.

As may be imagined, from what we have already said, his Lordship does not entertain a high opinion of the value of the medical certificate, as a *medical* certificate. His remarks upon this point are worth noting, as they show good reason for the greater cultivation of the study of mental disorders among medical men, but they also afford another example of his Lordship's inconsequent method of reasoning.

"197. *Sir Erskine Perry.*] But your Lordship does not consider this medical certificate of much intrinsic value, as the medical men know no more of lunacy than any other educated men in society?—In many cases they do not; if you were to look at many of these certificates, and read the reasons which are assigned by them, you would think that some of them were less capable than many others in society.

"198. Then what is the value of such a certificate?—Many of these medical men do not judge from their medical knowledge, but from their general experience of mankind. They see a person, and they see that he is mad; but some of them may give very bad reasons, although their conclusions may be just, for shutting him up.

"199. Then if it be as well to have the certificate from some educated man, a medical man is as good as any one you can get?—Yes; they will always ascertain whether there is any functional disorder.

"200. *Chairman.*] They are at any rate as good as any other men, for they possess knowledge which other men have not?—Yes. As I said before, the certificate has been on the whole very efficacious, and I think, as I said before, that not more than one or two cases have been admitted into asylums without a foundation."

The chief point with which we have to do in this quotation is the reiteration of the opinion that few unnecessary or unjustifiable certificates of insanity have been given by medical men. This may well lead to a doubt whether any benefit would result either to the patient or to his friends from requiring that any other precautions against improper confinement than those already in vogue should be had recourse to. Lord Shaftesbury states that the establishment of *medical examiners* who should examine and countersign the certificates within a brief period after a patient's removal to an asylum, was suggested by the Commissioners "*only as an expedient, with a view of satisfying public feeling, and not with any hope that it would really be effective*" (Qy. 81); and subsequently his Lordship states, "I do not see any necessity for it" (that is for the appointment of medical examiners: Query 823). But surely the public would be better satisfied with the facts and proofs, which the Commissioners apparently have at command, and which would show that individuals were already sufficiently guarded against being unduly confined, than with suggestions for useless and expensive legislation?

Mr. Bolden (Queries 2625—29) proposes to give greater security to the public through the magistrates. He suggests that no lunatic should be admitted into an asylum unless the certificates were accompanied by the warrant of a magistrate, who should have the power of satisfying himself of the necessity for the confinement. The sheriff exercises a judicial function of this kind in Scotland, but from the first Report of the Scotch Commissioners in Lunacy, we learn that from the exercise of this function, a serious impediment is often thrown in the way of the early treatment of cases, and that, indeed, the evils arising out of the arrangement far outweigh the good. The Commissioners do not, however, advise that the sheriff's authority in regard to lunatics should be entirely done away with, but that it should be so far modified as not to interpose any obstacle to the immediate reception of cases into asylums when requisite. The Scotch experience is in reality far from favourable to the exercise of a power such as that which Mr. Bolden would vest in a magistrate.

A suggestion of Lord Shaftesbury's, having reference to the prevention of unnecessary detention of lunatics, is worthy of consideration, and would seem to be well-calculated to give increased confidence to the public. He says :—

"201. * * * The only suggestion that I can make with regard to the certificate is this, that supposing the present system to go on, I think that some benefit would be gained by granting in the first instance a certificate for only three months; now it is granted in perpetuity, so long as the patient is under the disorder; but in the first instance, I would have it given for only three months, and I think the effect would be to compel a revision of the case by the family or friends; the relatives would then be obliged to look again into the matter, as they would know that in all probability, if they did not do so, the patient would be returned upon their hands.

"202. In order to justify the retention of a patient for more than three months, that form of certificate would have to be gone through again?—Yes, but only in the first instance; I should not have it every three months; I should say that it would be necessary at the end of the first three months after the detention that the certificate should be reviewed. That I know was the opinion of Dr. Heberden in 1815, when he gave evidence before the House of Lords."

His Lordship laid considerable stress upon the value of a power which would have been given to the Commissioners, by Mr. Walpole's proposed amendment of the Lunacy Law, and by which they might authorise the provisional discharge of patients from asylums for periods say, of two, four, or six months, the original certificates of insanity remaining in force during the interval. This power doubtless would prove of no small benefit.

The condition of single patients in private houses was entered into tolerably fully during Lord Shaftesbury's examination, and his Lordship spoke even more unfavourably of the position of lunatics so circumstanced than of those in private asylums. He asserted that he had reasons for the belief that many of the single patients, "of whom we (the Commissioners) know nothing, are in a very bad ('most frightful:' Qy. 354) condition" (Qy. 280); but when asked for the grounds of this belief, his

Lordship quoted examples from the year 1846—a period when the powers of the Commissioners in Lunacy had hardly come into operation, which powers were specifically designed, among other things, to remedy the then acknowledged ill-treatment of single patients! If now we turn to his Lordship's evidence as it bears directly upon the state of single patients at the present time, we find as follows :—

"352. *Chairman.*] In the cases that you have recently discovered within the last three years of single houses, where single patients were kept, and where a certificate ought to have been sent to you, but it was not, did you find the treatment particularly bad?—No, not particularly bad; in a few instances we found that there had been a good deal of neglect; the poor patient has been in a miserable isolated state, and very little attended to; in one or two instances they were very well taken care of; but in the vast proportion of instances, I should say, that there was a great deal of neglect, in consequence of people feeling that there was no supervision over them.

"353. It is more a want of sympathy, and the state of isolation in which they are, than any actual cruelty which you believe to be practised towards them?—Exactly so. We did not find any cases of special ill treatment, but we were obliged to take all our evidence from persons interested to give the most favourable representation of the case, while the poor patients could give no evidence in the miserable position in which they are placed, and Heaven alone knows how they are treated."

Here, then, it would appear that the only evidence his Lordship has to offer in support of his assertions of cruel neglect, are surmises grounded upon suspicions similar to those which formed the basis of his assertions respecting the "indefensible and abominable" character of private asylums. Of the value of these suspicions as evidence, his Lordship has given us the measure, by his frank confession as to their origin (*ante*, p. 384); but we may well ask what good is to arise from raking up events which occurred *many years ago*, and advancing them as illustrations of an *existing state of things*, when we are seeking to know the *present* condition of a class of lunatics, and the mode in which the law operates for their protection—that law having specific provisions to prevent the abominations which Lord Shaftesbury named, and having come into operation since they occurred?

His Lordship thinks that the law should be so extended that a large number of single patients, so-called *nervous* patients, and others not strictly lunatics, but almost verging on lunacy, should come under the jurisdiction of the Commissioners in Lunacy. The addition which his Lordship is desirous of making to the law is as follows :—

"432. * * * 'Whereas many persons suffer from nervous disorders and other mental affections of a nature and to an extent to incapacitate them from the due management of themselves and their affairs, but not to render them proper persons to be taken charge of, and detained under care and treatment as insane; and whereas such persons are frequently conscious of their mental infirmity, and desirous of submitting themselves to medical care and supervision, and it is expedient to legalize and facilitate voluntary arrangements for that object so far as may be compatible with the free agency of the persons so affected, be it enacted as follows:—

"'Subject to the provisions hereinafter contained, it shall be lawful for any duly-qualified medical practitioner, or other person by his direction, to receive and entertain as a boarder or patient any person suffering from a nervous disorder or other mental affection requiring medical care and supervision, but not such as to justify his being taken charge of and detained as a person of unsound mind.

"'No person shall be received without the written request in the form, schedule , to this act, of a relative or friend who derives no profit from the arrangement, and his own consent, in writing, in the form in the same schedule, the signatures to which request and consent respectively shall be witnessed by some inhabitant householder.

"'The person receiving such patient shall, within two days after his reception, give notice thereof to the Commissioners in Lunacy, and shall at the same time transmit to the Commissioners a copy of the request and consent aforesaid.

"'It shall be lawful for one or more Commissioners, at any time after the receipt of such notice as aforesaid, and from time to time, to visit and examine such patient, with a view to ascertain his mental state and freedom of action; and the visiting Commissioner or Commissioners shall report to the Board the result of their examination and inquiries.

"'No such patient shall be received into a licensed house.'

"So that we should say that every person, professional or not, who receives a patient into his house, or attends a patient in such circumstances, should notify it to the Commissioners; but we should not require them to notify it until after three months should have elapsed, because a patient might be suffering from brain fever, or a temporary disorder; but I would say that any person accepting or attending a patient in these circumstances should notify it to the Commissioners, after three months shall have elapsed from the beginning of the treatment.

"433. *Sir George Grey.*] Do you think that this would include a class already not included under the general terms of 'unsound mind,' the description being, 'Whereas, many persons suffer from nervous disorders, and other mental affections, of a nature and to an extent to incapacitate them from the management of themselves and their affairs.' Would not that definition almost be included already in the description of persons of 'unsound mind?'—The distinction is so very fine-drawn that it is almost impossible to say, for there are many persons in a nervous state, though not of unsound mind, and yet who cannot take the management of their own affairs.

"434. *Sir Erskine Perry.*] It seems to me rather an objection to that form of drawing the clause, that it makes it lawful for any medical man to do so and so, whereas, by the present state of the law, it is lawful for any person to receive any person into his house?—I should not wish to throw any impediment in the way of that; no impediment in the early treatment of cases of that kind; I only want, in giving facilities, to take care that there shall be no abuse.

"435. But it is lawful now for anybody to do it?—We might alter it; it is not drawn with sufficient technicality, no doubt."

It is needless, perhaps, to indicate any other objections to his Lordship's suggestion than those pointed out by Sir George Grey and Sir Erskine Perry; but we may add that we are much afraid it would be necessary to increase greatly the number of Commissioners, if their jurisdiction were to extend to all "nervous" cases, and it would be cruel and monstrous to class the nervous with insane cases and treat them as lunatics.

There is nothing in his Lordship's observations upon criminal lunatics which requires particular notice, but an incidental commentary upon the plea of insanity in criminal cases is deserving of regard:—

"I am unwilling to say, [remarked his Lordship, in reference to the life-confinement of persons who have committed great crimes under the influence of insanity] that in every instance a man should be condemned to imprisonment for life because he had committed, however fearful an act in a sudden aberration of mind. That must be left, I think, to the discretion of the Secretary of State; but then it is becoming more delicate and complicated every day, because, it will be observed, that the medical men who are called to give evidence, in 99 cases out of 100, give evidence in favour of insanity, *and persons who have any great crime to perpetrate know that very well.* I am told that there is one medical man of considerable reputation, who has openly said that it is his rule always to give evidence in favour of insanity, as he has such an opinion of the general misconstruction of the whole human mind; and even when it is not so, see how juries are influenced by the profession. They bring in other arguments which perplex the jury very much, who are told that the man is not absolutely insane, but that he is labouring under the effect of an impulse that he cannot control. I must say that the medical men oftentimes talk an immense amount of nonsense when they come before juries, and I believe that that is the received opinion."—(p. 52.)

We presume that as his Lordship's suspicions and assertions respecting private asylum proprietors were dictated by the consciousness (as he avowed) of what he would do himself if he were in their position; so the last sentence in the preceding quotation was dictated by a consciousness of the nature of his efforts in the preceding portion of his examination to enlighten the committee respecting the nature of insanity. As to his assertion respecting the particular medical man mentioned in the quotation, it is evident that his lordship has been victimized by a *canard.* We can quietly submit to an implication which involves judge, jury, and the bar in a very comprehensive charge of ignorance; but what shall we say to the grave assertion that the plea of insanity, as supported by medical men, is an incentive to great crimes? The assertion is either true or not true. If it be true, there must be evidence in support of it. Where is the evidence? If it be not true, how must we characterize so unjustifiable an assertion? The remainder of the paragraph in which the statement is found points too clearly to the kind of evidence (if mere unsupported assertions can be termed evidence) on which Lord Shaftesbury would rest his statement, and as clearly to the opinion which every right thinking man must entertain of it.

On one of the most important portions of his Lordship's evidence, that on the condition of lunatics in workhouses, we shall not dwell, as we have elsewhere noticed at length the recent Report of the Commissioners in Lunacy on that question, and his Lordship's statements were based upon the facts contained in that Report.

A few additional remarks will suffice to bring our notice of Lord Shaftesbury's evidence to a conclusion.

His Lordship urged the importance of raising the character of the attendants who had charge of lunatics, and the necessity that they should receive good and sufficient wages, in order that an intelligent and trustworthy class of people might be secured; he also suggested that the superannuation of attendants in public asylums should be placed upon a more definite, and in other respects, better footing. He would fix the minimum number of attendants in these asylums at one to every twenty-five patients.

In his remarks upon private attendants his Lordship contrived to introduce a charge against medical practitioners in lunacy, which cannot well be passed unnoticed, but which serves only as another example of the incautious method in which his Lordship's assertions when affecting medical men were made. The character of the assertions will be sufficiently understood by the questions founded upon them in Dr. Sutherland's examination:—

"2135. I will read this statement to you, 'Several of the London physicians, practising in lunacy, conduct a regular trade in the supply of attendants to medical men and others. They pay them a yearly stipend, and support them when they are not employed; when they are employed, the physician takes from two-thirds to three-fourths of the attendants' fees for his own profits?'—I think I had better state exactly how it is; the attendants have a regular salary, and when they go out, they are on the principle exactly of the Nursing Sisters' Institution of Queen's Square, they have double salary, that is, they have fifty guineas instead of twenty-five, and then a sum is certainly given to the asylum they come from, for their board and maintenance, when they return to the asylum.

"2136. *Sir George Grey.*] Do you mean that the attendants themselves receive fifty guineas, when actually employed, instead of twenty-five?—Yes.

"2137. Then what is it that goes to the asylum?—A guinea a week.

"2138. Paid by the attendant, and deducted from the fifty guineas? —No; when they are out, the attendants have a guinea and a half, besides the salary which they have, which is twenty-five guineas a year, they have that half guinea a week per year.

"2139. Is that paid by the proprietors of the asylum?—No, it is paid by the friends who employ them.

"2140. And that goes to the asylum?—A guinea a week goes to the asylum.

"2141. *Colonel Clifford.*] Is it the fact that some of these attendants are in the apparent position of owners of the houses in which the patients are, and that when in that apparent position they pay over a portion of the sum paid for the maintenance of the patient to any medical man?—I believe it was so in former days; and when I commenced practice, I was asked to do it, but I always refused, and in fact I never have received anything but my own professional fees, as any other medical man would receive for attending on his patient.

"2142. You are not aware whether any other medical man has acted on that same principle, and refused?—I do not know.

"2143. *Sir George Grey.*] Is it the case with regard to those payments, when one of these attendants is employed in the service of any family, that he receives from the family a guinea and a half a week, a guinea of it going to himself, and half a guinea to the proprietor of the house?—Just the contrary; the half guinea goes to him, and the guinea to the asylum.

"2144. Does he receive that half guinea in addition to the twenty-five guineas a year salary?—Yes.

"2145. *Sir Erskine Perry.*] Then the salary from the family must be seventy-five guineas a year, and not fifty guineas?—Yes. It is supposed that the proprietors of asylums derive a large profit from this plan, but I know from calculation that it is not so, and that it is done at a loss, when you calculate the wages and the board and lodging of these attendants.

"2146. Can you give the Committee anything like an average of the time during which these attendants are out of employment?—They are generally thrown out of employment in the winter. They are employed more in the spring.

"2147. With regard to the former practice that existed, as to sharing the allowance paid for maintenance with the medical man, you do not know whether the same practice exists now, or have you any reason to believe that it does?—I really do not know."

Lord Shaftesbury's assertions were grounded upon the letter of an unnamed "medical man of great experience in insanity" received on the morning of the second day of his Lordship's examination. Has his Lordship made any inquiry as to the correctness of the statements contained therein? For our own part, we were not aware until we read Dr. Sutherland's answer to query 2141, that the custom there indicated had ever existed; and certainly an example never came under our own observation. But surely we have a right to expect that if statements of the kind referred to are made, that the Commissioners in Lunacy should take at least ordinary trouble to ascertain whether they are correct now or not, or whether they were correct only many years ago, and that such statements should not be mentioned on the irresponsible authority of an unnamed correspondent.

In his remarks upon the Bill, laid before the late Parliament, "to Amend the Laws concerning the providing Lunatic Asylums for Counties and Boroughs, and the Maintenance of Pauper Lunatics," Lord Shaftesbury approved of the power proposed to be given by the Bill to the Secretary of State to effect an annexation between counties and boroughs, without the consent of the Committee of Visitors of the county asylums, as well as to direct that the boroughs provided with accommodation for their lunatics in county asylums shall pay an equitable sum towards the maintenance of such asylums. His Lordship also approved of the remaining sections of the Bill, to wit, that plans, &c., of visitors when not approved by the Quarter Sessions should be submitted to the Secretary of State; that land and buildings may be taken from year to year, on lease, by way of addition to an asylum; that estimates are to accompany the plans which are to be submitted to Commissioners under 16 and 17 Vict. c. 97, s. 45; that visitors of lunatic asylums may contribute to expenses of enlarging or providing burial-grounds; that s. 132, c. 97, 16 and 17 Vict., be repealed in respect to the signification of the word "county" therein (to meet a difficulty which has occurred at Norwich); that a patient may be ordered to be discharged if defective medical certificate be not amended within fourteen days; and that the provisions for the chargeability of pauper lunatics whose settlements cannot be ascertained when found in certain boroughs, 18 and 19 Vict., c. 105, s. 14, be repealed, and other provisions, making such paupers chargeable upon the said boroughs be enacted, as set forth in the bill.

In the course of his remarks, Lord Shaftesbury made one or two suggestions which may be noted. He expressed a desire that a clause should be added "making it part of the duty at the Quarter Sessions to read openly in court the 'entries' which are made by the Commissioners at their several visitations once a year"

(qy. 759). He expressed the opinion that the payment of the medical superintendents of county asylums was much too low, and that he hardly knew an asylum where the salary was adequate to the work done. In this we fully concur with his Lordship, as also in the subsequent remark, "I cannot think that any superintendent ought to receive much less than 500*l.* to 600*l.* a year, besides a house and allowances" (qy. 767). His Lordship expressed in detail his opinions upon the superannuation of attendants and medical officers, affirming that it should be fixed and not left optional to the visitors; and finally we may note that his Lordship considered that the Secretary of State's power should be increased in reference to the enlargement of public asylums, so that some check might be placed upon the indefinite and enormous increase of several of the large county asylums.

His Lordship's remarks upon the Bill to "Amend the Law concerning the Care and Treatment of Lunatics," require only brief notice, as we have already, as will be immediately seen, considered or glanced at the principal points in his Lordship's evidence which touch upon the provisions of the Bill, and require attention.

The first clause of the Bill provides that a licence shall not be granted by Justices of a house for the reception of lunatics without inspection and Report by the Commissioners. This would not supersede the action of the local magistrates, but would give them the benefit of the Commissioners' experience, and is so far good. The second clause provides for an alteration in the form of the licence, which the first clause would necessitate; and the third clause provides that notice of all additions and alterations in licensed houses should be given to the Commissioners. Clauses four to nine inclusive, relate to the appointment of medical examiners; and clauses eleven to fourteen inclusive, detail the duties of the medical examiners, and the mode in which they are to be effected. We have already seen from Lord Shaftesbury's statements, that the appointment of these examiners would probably have no other benefit than that of satisfying public feeling. The tenth clause provides that the copy of order and certificates shall be sent to the Commissioners within twenty-four hours. The 15th clause provides that certain persons are not to sign certificates for the reception of patients, the said persons being the proprietors of or having an interest in licensed houses. This, as we have already remarked, would deprive the public, as a rule, of the most trustworthy, because the most experienced, opinions upon cases of lunacy under circumstances where the said opinions are of most moment, to wit, when required to be stated in a certificate of insanity. The 16th clause provides that a patient may be ordered to be discharged if a defective medical certificate be not amended within fourteen days; the 17th provides that patients may be permitted to be absent on trial from hospitals and licensed houses; the 18th provides that notice of the recovery of a patient shall be given forthwith to the Commissioners; the 19th, that every house licensed for fifty patients or more, shall have a resident medical practitioner; that every house licensed for more than thirty, and less than fifty, and having no resident medical man, shall be visited *daily* by a legally qualified practitioner; and every house licensed for less than thirty, shall be visited by one *thrice* a week, if a medical man be not resident; the 21st clause defines certain powers to be exercised by the Commissioners or visitors to require more or less frequent medical visitation if necessary; the 22nd clause provides that licensed houses shall be visited by a single Commissioner once a year in addition to the visits now required, and that every single Commissioner visiting alone shall have the like powers as two Commissioners would have under section 61, 8 and 9 Vict.; the 24th clause provides that the Commissioners' shall report specially on cases admitted within the year preceding each visit; the 25th provides that copies of Commissioners' entries in the books of provincial licensed houses shall be sent to the clerk of the visitors; and the 26th clause provides that the proprietors of licensed houses, and persons having charge of single patients, shall furnish information to the Commissioners as to payment for patients.

Lord Shaftesbury approves of the whole of the clauses, qualifying, as we have seen, his approval respecting medical examiners. His Lordship would, however, add a clause to prevent corrupt agreements between medical men and the proprietors of asylums; another to make it "compulsory" upon every medical man receiving a patient termed a nervous patient, or any medical man attending a patient called a nervous patient, to communicate the same to "the Commissioners" (qy. 921); and also a permissive clause respecting the construction of "middle-class asylums."

To the objections we have already made respecting certain clauses of the Bill, we would add solely a remark respecting the 26th clause. This clause does not provide that on due and sufficient cause being shown warranting the suspicion of neglect of a patient, the Commissioners shall have the power of requiring an account to be submitted to them of the receipts from and expenditure for the patient, but gives them an unlimited power of requiring such accounts to be laid before them, when, how, and for whatever cause they might think fit! Such a proposition as this evidently emanates from the same suspicious spirit which infects the whole of Lord Shaftesbury's evidence respecting medical men practising in lunacy. It is not a *bonâ fide* provision, and would, no doubt, if it were enacted, have the same fate as all vexatious legislative provisions; but, in the meantime, it, and the rest of the clauses of the Bill conceived in the same spirit, would have a diametrically opposite effect to that which they were intended to have. It is surely sufficiently disheartening to the medical practitioner in lunacy to be assailed by foul suspicions which are unsupported by almost a tittle of trustworthy evidence, and which by their vagueness elude all attempts thoroughly to crush them by an appeal to facts; but when these suspicions are made grounds for legislation, it is clear what must follow. No man of right feeling, honour, and integrity will expose himself to the annoying and harassing consequences which such bungling and disgraceful legislation would certainly entail upon him; every man who might be exposed to such consequences, but who had any respect for himself, would as soon as possible escape from the questionable, suspected, and degrading position in which he would be placed; and, as a consequence, the treatment of the unhappy insane would be entirely left to inexperienced persons, and to those with whom mercenary considerations might outweigh all feelings of personal honour. Thus we should have brought about the very state of things implied by the suspicions expressed by Lord Shaftesbury; there would be a distinct retrograde movement in all that referred to medico-psychological science, and the care and treatment of lunatics; and the present attempt to benefit the insane by throwing about them more effective legal protection, would become (as it bids fair to be) a *quasi*-philanthropical fiction.

One would, *à priori*, conceive, that all legislative enactments regarding the care and treatment of the insane, should have in contemplation one great essential and important object—viz., to encourage medical men of reputation, practical experience, and of high moral character, to identify and connect themselves with institutions organized for the reception and cure of lunatic patients, by placing confidence in the honesty and integrity of their conduct, and legally protecting them, whilst in the discharge of these trying, anxious, and responsible duties, from all unfair and unjustifiable attacks and aspersions. Alas! a course the very opposite of this appears to be the one held up for popular admiration and legislative adoption. The design appears to be to *drive* men of character, respectability, and honour out of the speciality, by rendering their position so odious, offensive, and repulsive, that no gentleman who had any respect for himself would think of occupying it. For the misdeeds of one or two delinquents, the whole body of men connected with asylums are made to suffer! The order of the day is, hunt the "mad-doctors" down! extend to them no mercy, give them no quarter! treat them and legislate for them as a *suspected class!* Is it right, honest, just, and consistent with English notions of fair play, thus to join in the hue and cry against a section of the medical body who, at great pecuniary and personal sacrifices, engage themselves in so thankless, but nevertheless if rightly viewed, honourable and sacred vocation! "Oh," says the Earl of Shaftesbury, "the system of profit vitiates the whole proceeding!" In reply to this disingenuous remark, we ask, whether the bulk of men connected with, and having an interest in, private asylums, are not far above such low mercenary and commercial considerations!

If a physician is, alas! by nature so dishonest, corrupt, and so incapable of being influenced by other than sordid and selfish motives, he has ample opportunities of exhibiting his character in *ordinary* as well as in *special* departments of practice. But is there a man in the ranks of a profession so honourable as that of medicine, who would be guilty of such brutal and villainous conduct? We maintain without fear of contradiction, that there is no kind of justification or excuse for the imputations made against the respectable class of men engaged in this country in the treatment of the insane. It was asserted by the Earl of Shaftesbury, that patients are detained in asylums for considerations of profit longer than their state of health justified! But we ask, upon what *data* does his lordship base so serious an imputation? Who is the best judge, whether a patient has sufficiently recovered to warrant his discharge from control and medical treatment and supervision, the Commissioners who only see him for periods ranging from ten to thirty minutes, four times in the course of the year, or the medical superintendent of the asylum who has the case constantly under his observation, and who is most anxious to send him back into the world cured? Is it not a notorious fact, that a large number of patients who are temporarily removed from asylums return to them shortly afterwards in an acute state of insanity? What object is gained by thus sending them from the institution? Is not this course productive, in many instances, of sad results, by seriously interfering with a regular and continuous course of treatment, upon which the cure in the majority of cases depends?

If a physician discharges his duty, and says a particular patient is not yet in a condition to leave the asylum, that he is not sufficiently well to justify the experiment of his removal even into lodgings, he unavoidably exposes himself to suspicion, and gives his *enemies* the opportunity of saying that he is detaining the patient for his own pecuniary advantage!

The psychological physician, it must be admitted, has not even fair play in this country. In France, Germany, and other continental countries, as well as in America, men of the highest professional standing, of unimpeachable honour, of unsullied character, and of great scientific eminence, do not consider themselves guilty of an act of social or professional degradation by associating themselves with private asylums for the insane, and even in deriving what Lord Shaftesbury terms, a "profit" for their care and treatment. Every labourer is worthy of his hire; and surely the man who occupies his talent, professional skill, and time in restoring the poor lunatic to the bosom of his family in a state of sanity, cannot be overpaid for the great benefit he has thus conferred. These matters are not to be estimated as you would the value of a horse or a piece of land. It is impossible for the Commissioners, or any other body of men, to establish any scale of remuneration for such cases. How is it possible for them to strike a balance, and say such a patient costs so much, and then estimate to a fraction the profit which the proprietor of the asylum puts into his pocket? Loose calculations like these are utterly valueless and worthless apart from a knowledge of the particulars connected with special and peculiar cases. One patient may pay a sum of money apparently large and quite disproportionate to the accommodation afforded; but there may be circumstances in connexion with such a case known only to the parties immediately connected with it, which renders it, instead of being profitable, an actual loss to the medical man. Such is occasionally the case. If it were a mere question of rooms, chairs, tables, curtains, pictures, beef, mutton, and wine, the question would be amenable to ordinary commercial considerations and calculations; but there are other and more important elements that enter into the question, and which remove it entirely from such a basis. Is not the skill bestowed in the treatment of the case to enter at all into the calculation when the question of "profit" comes to be considered? Are not the wear and tear of body and mind, the constant fret, worry, anxiety, and *occasional loss of reason* to which the physician is exposed who has under his care this unhappy class, points to be estimated when making out the balance-sheet?* Surely it is monstrously unjust to ignore this view of the matter in any calculations that may be made as to "profit and loss" in any asylum or particular case!

Really one would conceive, after wading through the bulky Blue Book, that a lunatic asylum was a perfect Elysium, a paradise upon earth, and that the physician who had the charge of it occupied a most enviable, agreeable, and pleasing position! In

* We could mention the names of several physicians who have become insane in consequence of anxiety of mind caused by residing in lunatic asylums, and constantly associating with the insane.

such an institution, where the spirit of love, concord, and harmony reigned paramount, he would, surrounded by his happy and contented family, be exposed to no care, trouble, and anxiety. If one of his associates were from a mistaken impression or an erroneous judgment, to try to fracture the proprietor's skull, it would be considered an incident hardly worthy of notice. If another lively companion, whilst enjoying the social privileges of dining with the proprietor and his family seized hold of a knife whilst at dinner, and were deliberately to cut his throat from ear to ear, in the presence of the domestic circle, thus deluging the table with blood, it is a mere *bagatelle*, not entitled to one moment's consideration.* If another skittish associate concealed about his person for some weeks a deadly weapon, with the avowed intention of taking the first favourable opportunity of waylaying the medical superintendent, and estimating the distance between the cutis and the right or left ventricle of his heart;† if another piece of eccentricity howled like a wild animal over his head for six consecutive hours, rendering "night hideous," and all attempts to "steep the senses into forgetfulness," abortive, these would be nothing but pleasantries and pleasures, making life happy and agreeable, and a lunatic asylum the most charming residence that the imagination could by possibility picture to the mind!

As to the proposed clause respecting *nervous* patients, we shall be anxious to know how Lord Shaftesbury will define the term *nervous*. When this is done satisfactorily, then perhaps the clause will be practicable.

Lord Shaftesbury's evidence guided, in a great measure, the committee in the examination of subsequent witnesses; but with his Lordship's evidence terminates the necessity for any further detailed notice of the statements laid before the committee. Mr. Gaskell, one of the medical commissioners in lunacy, gave additional evidence on the duties of the commissioners, and on the condition of the lunatics in workhouses; and Mr. Farnall, the Metropolitan Inspector of the Poor, gave also evidence on lunatics in workhouses. Mr. F. Barlow, one of the Masters in Lunacy, and Mr. Wilde, the Registrar in Lunacy, gave evidence on the duties of the Masters and on Chancery lunatics. Dr. H. H. Southey, one of the Medical Visitors in Lunacy, also gave evidence on Chancery lunatics. Evidence was given by Mr. Johnson, one of the Visiting Justices of the Surrey County Asylum; by Sir A. Spearman, the Chairman of the Committee of Visitors of Hanwell Asylum; by Mr. Cottrell, the Chairman of the Committee of Visitors of Colney Hatch Asylum; by Mr. Woodward, the Chairman of the House Committee at Colney

* This circumstance occurred in a private asylum near London. † Ditto.

Hatch; and by Sir G. Robinson, one of the magistrates of the county of Northampton, on the condition and general management of the insane in lunatic asylums. Dr. Conolly and Dr. J. Sutherland, both gave important evidence before the Committee, that of the former forming a grave and weighty protest against the unguarded statements of the Chairman of the Commissioners in Lunacy, and against unnecessary and vexatious legislation; and that of the latter is very valuable from the light which it throws upon the condition of single patients in private houses. Dr. Hood, Col. Jebb, and Mr. Everest of the Home Department, gave evidence upon the condition of criminal lunatics. Dr. Hood expressed the opinion that criminal lunatics would be better treated in special wards connected with convict prisons, than in a special asylum such as is now being erected for them by the State; but Col. Jebb (qy. 3293) thought such a course highly inexpedient. Mr. Monckton Milnes, M.P., gave brief evidence respecting certain lunatics in York Gaol; and lastly, Mr. Gilbert Bolden, and Admiral Saumarez, gave evidence as representatives of the "Alleged Lunatics Society." Mr. Bolden makes many suggestions extending over almost the whole field of lunacy legislation, and his suggestions merit attention, although they are too frequently based upon evidence insufficient to show their necessity, and they partake too largely of that perverse, single-eyed attention to the *civil* rights of lunatics, as contradistinguished from, and to the neglect of their *medical* rights, which is too common with lay lunacy-law reformers. He sees a greater evil in a person being too hastily confined as a lunatic, than in throwing impediments in the way of the early treatment of incipient cases of lunacy. It is, however, the avoidance of both these evils which should form a chief question in lunacy legislation, but which has, thus far, been entirely omitted from the consideration of the Select Committee. *How shall we best secure the necessary treatment of incipient cases of lunacy in private asylums, or "middle-class" asylums if established, without branding the cases from the outset as lunatics?* This is the cardinal point for the nascent lunatic, and the first and main consideration of the medical man; but it is just this point that we cannot get the lay-friends of lunatics to listen to. We feel convinced that the question can be solved if the public will rest content for a short time to regard medical practitioners in lunacy as curative agents, and not as avaricious ogres, such as Lord Shaftesbury portrays them; and we also feel assured that the question might be solved with due and strict attention to the preservation of the civil rights of the lunatic.*

* See an important suggestion, bearing upon this question, by the Scotch Commissioners in Lunacy, pp. 434 and 437 of the present number of this Journal.

In bringing our notice of the Select Committee's Report to a close, we may remark that, if in reading the minutes of evidence we note the details, (where those details are simply records of facts,) which indicate the manner in which the existing lunacy laws operate, we may gather much valuable information; if we note the spirit which characterizes those portions of the lay-evidence which may be assumed to represent the public desire for further legislative interference respecting lunatics, we cannot but regard it with profound regret, and entertain a fear that it will impede rather than promote effective legislation, or if it does hasten legislation forward, will so affect it in certain most important particulars as to complicate the whole matter still more; and if we note (which most concerns us at the present moment) what solid material the voluminous evidence affords us to aid in the formation of a firm foundation for further legislation, we are constrained to exclaim, "O monstrous! But one halfpennyworth of bread to this intolerable deal of sack!"

The conclusion of the whole matter is precisely that which the Committee came to, that the evidence is not sufficient to enable any one to form a definite opinion on the subjects to which it referred, and that "the inquiry is altogether incomplete."

---

